# A Connected Community Approach: Citizens and Formal Institutions Working Together to Build Community-Centred Resilience

**DOI:** 10.3390/ijerph181910175

**Published:** 2021-09-28

**Authors:** Blake Poland, Anne Gloger, Garrett T. Morgan, Norene Lach, Suzanne F. Jackson, Rylan Urban, Imara Rolston

**Affiliations:** 1 Dalla Lana School of Public Health, University of Toronto, Toronto, ON M5T 3M7, Canada; norene.lach@mail.utoronto.ca (N.L.); suzanne.jackson@utoronto.ca (S.F.J.); imara.rolston@utoronto.ca (I.R.); 2 Centre for Connected Communities, Toronto, ON M1E 2S2, Canada; agloger@connectedcommunities.ca; 3 Department of Geography & Planning, University of Toronto, Toronto, ON M5S 3G3, Canada; garrett.morgan@mail.utoronto.ca; 4 Institute for Management & Innovation, University of Toronto Mississauga, Mississauga, ON L5L 1C6, Canada; Rylan.urban@mail.utoronto.ca

**Keywords:** resilience, community resilience, community development, public health, connected communities approach, emergency preparedness, governance

## Abstract

Urban resilience research is recognizing the need to complement a mainstream preoccupation with “hard” infrastructure (electrical grid, storm sewers, etc.) with attention to the “soft” (social) infrastructure issues that include the increased visibility of and role for civil society, moving from (top-down, paternalistic) government to (participatory) governance. Analyses of past shock events invariably point to the need for more concerted efforts in building effective governance and networked relations between civil society groupings and formal institutions before, during, and after crisis. However, the literature contains little advice on how to go about this. In this paper, we advance a Connected Community Approach (CCA) to building community resilience with a specific focus on the relationship between community and formal institutions. In the literature review that informs this work, we assess the current, limited models for connecting communities to formal institutions, as well as the emerging role of community-based organizations in this work, and we offer our own assessment of some of the key tensions, lacunae, and trends in the community resilience field. Principally, we explore the potential of the CCA model, as spearheaded by the East Scarborough Storefront and the Centre for Connected Communities in Toronto, Canada, as a promising approach for building the relational space between civil society and the state that is so often called for in the literature. The paper concludes with future directions for research and practice.

## 1. Introduction

Resilience is a key feature of healthy, vibrant cities [1,2,3,4,5,6,7]. Despite the recent exponential increase in scholarship on resilience, critical gaps remain in our understanding of what, why, and for whom resilience manifests in our communities [8]. While much attention has been paid to resilience at the individual, organizational, and institutional levels, the need to build community resilience in the face of climate change and extreme weather is becoming more widely acknowledged [9,10,11,12], alongside more recent attention to resilience in the face of pandemics [13,14,15].

Community resilience foregrounds the role of communities in responding, recovering, adapting, and transforming before, during, and after crises. To build resilient communities, the dominant institutional approach tends to favour top-down initiatives led by professionals trained in emergency preparedness and response. However, historic and recent community-led responses have brought to light the need for communities themselves to be key actors in both short- and long-term resilience strategies [10]. This need is underscored by retrospective analyses of emergency response and recovery in post-Katrina New Orleans [16], the aftermath of Hurricane Sandy [17,18], and extreme weather events in the Appalachians [19] and High River, Alberta [20]. Such events demonstrate the critical role of grassroots efforts in the immediate aftermath and longer-term recovery of communities post-emergency, as well as the ways in which formal response systems (once they do activate) can neglect or run roughshod over grassroots community work and squander critical opportunities for more constructive collaboration.

Addressing the role of the community in building community-centred resilience begs the question of “whose resilience” and “what kind of resilience”. In our view, community-centred resilience means investing in communities, as opposed to downloading responsibility to communities to fend for themselves.

## 2. Methodology

Building on two decades of research on community action (B.P., S.J.), including studies of how formal institutions such as hospitals [21,22,23] and public health units [24,25] work with community groups to address broader determinants of health equity (see also [26]), as well as two decades of work on the front lines of community development with marginalized groups undertaken by the East Scarborough Storefront (A.G.), resulting in the creation of the Centre for Connected Communities (C3; https://connectedcommunities.ca/, accessed on 18 September 2021), our community–university partnership obtained funding from the Canadian Institutes for Health Research (PCS-164954) to undertake a review of the community resilience literature with special emphasis on the role of civil society (in the context of relations with formal institutions) in the building of community-centred resilience.

We conducted a scoping literature review to explore the ways in which bottom-up, grassroots communities mobilize in response to shock events to address pressing community needs and community-defined goals, while also understanding how formal response systems can neglect or suppress these efforts. We developed key search terms and phrases based on earlier scoping reviews [27,28], key texts in the broader resilience literature [4,10,11,29,30], and emerging work that explicitly approaches resilience through an equity lens [31]. We used keyword Boolean phrases to conduct a title/abstract search in four distinct interdisciplinary databases: SCOPUS, OVID, ProQuest, and EBSCO. We further filtered results by those available in English. We then filtered results to focus on literature in Global North contexts, based on the assumption that such findings would be most relevant to resilience-building efforts in Canada. We acknowledge that there is a need for further research on the differences between community-centred resilience literature in the Global South, especially literature published in languages other than English, but that research stream is beyond the scope of this particular undertaking.

Our primary search was limited to results between 2010 and 2020, in an effort to capture the most recent scholarship on community-centred resilience building but acknowledge that the extended timelines and rigour of the peer-review process may result in a gap between current community-centred resilience work and scholarly publication. Our targeted search produced 12,791 results, drawn from a wide variety of disciplines, including geography, psychology, community development, risk management, disaster preparedness and response, civil engineering, urban planning, public health, management, public administration, and environmental science. After eliminating duplicates, we conducted a detailed title review of the remaining results to screen out sources that did not relate to community resilience building theory or practice.

To further refine our search, we screened out sources that explored resilience building at the individual level (psychology), economic (business continuity), and hard infrastructure (utilities, highways, wastewater treatment plants, public transit, IT networks) based on title and abstract review. Given our focus on community-centred resilience in urban Global North settings, we also screened out sources that focused primarily on community resilience in low-resource, Global South settings.

For grey literature, we identified sources using a combination of similar Google search terms and the snowball sampling method. We began by examining reports published by known organizations and localities active in the resilience space (e.g., 100 Resilient Cities, ICELI, The Kresge Foundation). Our civic and community partners also provided sources that they considered fundamental to their work. The primary and secondary sources identified in this initial literature scan were explored to identify commonly referenced academic and grey literature sources. Our final selection of 172 sources for in-depth analysis was reviewed by several members of the team, who are content experts in the field of community-centred resilience.

The following research questions informed our search strategy:

- What is the current understanding of what community resilience is, including both bounce back and bounce forward?
- What is the role of the community in building community resilience?
- What has been written about the need for connecting community and formal institutional players in building community resilience, and how has that been executed?
- What is core to the Connected Community Approach that is critical to fostering resilience in communities? What is adaptable based on context? How can the inherent values in CCA be operationalized in other contexts?
- How has resilience been measured? (This question is out of scope for this paper.)

Our intention with this paper is not to offer a comprehensive literature review on community resilience, but rather to highlight what that literature flags in terms of the social infrastructure required for effectively preparing for, responding to, recovering from, and bouncing forward from shocks, with a particular emphasis on the role of civil society and the relationships between community groups and formal institutions. Further, aware of the lack of guidance in the literature in response to repeated calls for better collaboration between community groups and formal institutions, we sought to make the case for a Connected Community Approach, especially as it relates to building resilience with racialized and marginalized communities, in the context of wider systemic drivers of persistent inequity, of which formal institutions are often at least complicit.

## 3. Framing Resilience as Social Infrastructure

An early discovery—and frustration—for many wading into the resilience literature is the multiplicity of definitions and perspectives that populate this space, with some scholars arguing that resilience is “too abstract to be meaningfully applied” [32] (p. 239). Scholars have shared frustration about the lack of clarity surrounding the term for two primary reasons. First, without a common definition, authors often talk at or around each other in siloed disciplines, without realizing that shared terms have very different disciplinary meanings [33,34]. Second, it is argued that a mutable definition makes it possible to claim any activity as building “resilience” without necessarily undertaking needed changes to existing structures, practices, and assumptions [35,36,37]. Thus, in practice, building urban resilience is often conflated with conventional forms of emergency preparedness that prioritize individual, household, and city-wide physical infrastructure, such as energy grids, stormwater management systems, and other civic and private sector assets, while ignoring equally essential dimensions of social infrastructure [38,39]. Thus, we maintain that it is important to explore how varied actors in a diversity of sectors and contexts can (re)conceptualize and (re)operationalize resilience [40]. Our emphasis here is on the often-overlooked social dimensions of community infrastructure that are increasingly recognized as essential to urban resilience [2,7,12,15,33,41,42,43,44,45,46,47,48,49,50,51].

Resilience was originally described by ecologist C. S. Holling [52] as the ability or capacity of an ecosystem to survive and withstand multiple external shocks without losing its fundamental functions or identity [37,53,54]. Resilient systems were understood to be capable of self-organization, learning, and adaptation [53,55], unfolding over the course of an adaptive cycle that includes four stages of change observed to be common to most ecological systems: growth, conservation, creative destruction, and reorganization [11,53,56,57].

Socio-ecological resilience emerged from the understanding that social and ecological systems are explicitly intertwined and must be considered together, rather than as separate distinct entities [58]. Within the broader field of social-ecological resilience, attention to dimensions of social resilience has explored how people collectively shape resilience [37,41,42,59], including the role of institutions [43]. Recent hurricane events in the United States and Puerto Rico (Katrina, Irma, and Harvey) [44,60], extreme weather in Appalachia [19], and Superstorm Sandy in the Greater New York City area [45,61,62] have drawn attention to the crucial role that racialized and low-income communities struggling with decades of disinvestment, poverty, racism, inequality, and other systemic and chronic stressors have played in responding to and recovering from shocks in the midst of ongoing chronic stressors, and how formal response systems often further exacerbate pre-existing inequities [16,37,63,64].

In contrast to early emphasis on hard infrastructure, more recent conceptions of community resilience have drawn attention to the collective role that people (and institutions) play in building or undermining resilience before, during, and after a crisis [38,65]. This branch of the literature has especially focused on social capital and the role of social networks in bonding and bridging community members, as well as linking communities to institutions and those in positions of power [46,47,48,49,50,51]. Social capital has been identified as a foundation for community resilience equal in importance to material and financial resources [7,19,44]. More recently, asset-based community resilience has been part of a broader shift towards equity, with the goal to address the inequitable impacts of shocks and stressors faced by communities that have been historically marginalized [38]. In this context, concerns have been raised about the ways in which discourses of resilience, couched in a language of celebrating community capacity and empowerment, can and have been used to download responsibility from the state to communities, who are expected to respond with volunteerism, mutual aid, collective goodwill, and the mobilization of community assets [38,62,66,67,68,69,70,71,72], although others claim that, from a postmodern perspective, the diversification and extension of engaged stakeholders holds the potential to upend existing narratives and power relations [73].

The downloading of responsibilities is especially pernicious in the context of the current neoliberal political environment of fiscal constraints and austerity, which often undercut the very capacities and components of communities and individuals which have been shown to support resilience [36,74]. Such appeals conveniently sidestep discussion of the systemic drivers of inequity that undermine community resilience and that exacerbate inequity and environmental injustice, as well as chronic disinvestment in racialized and low-income neighbourhoods. They also deflect attention from the egregious lack of connection of formal emergency response systems to the voices, needs, and aspirations of marginalized communities, as well as the expertise and capacities inherent in community systems of informal care and kinship. In our view, these represent tragic failures of opportunity for the co-production of effective responses to shocks and stressors that could combine the best of what both communities and formal systems have to offer.

Resilience is often framed as the capacity to bounce back to “normal” after a shock event without questioning the desirability, equity, or social and ecological sustainability of said “normal” [75,76]. When faced with shocks and stressors, definitions of resilience typically describe the recovery of systems to a pre-shock status quo, drawing upon ecological principles of equilibrium as a presumed ideal natural state [32]. Jon and Reghezza-Zitt [73] contrast this “managerial” approach with what they call an “adaptive/organizational” approach (focused on continuity of service), and a “progressive/transformational” approach that valorizes local knowledges, community capacity, and addressing inequities. This latter “bounce forward” approach is said to embrace shocks as an opportunity for transformational change, to better reflect post-shock realities, and/or to respond to a progressive vision of a more equitable and sustainable order [6,75]. However, as Naomi Klein reminds us in *The Shock Doctrine* [77], shocks can and have served as (sometimes intentional) opportunities for an entrenchment of vested interests and expansion of what she calls “disaster capitalism”. The austerity measures that followed the unprecedented public bailout of banks and financial institutions after the 2008 financial crisis is a case in point, as are current debates about the nature of a post-pandemic “return to normal”. As Blythe et al. [78] point out, there is a “dark side” to the mainstreaming of discourses of “transformational change” when it is rendered apolitical, inevitable, and assumed to be universally good.

Beyond shocks, “bounce forward” resilience calls for greater attention to the underlying stressors that undermine a system’s overall resilience [6]. Social resilience describes the “coupled, interdependent, and co-evolving” nature of stressors [58] (p. 7), such as poverty, environmental degradation, lack of social services, structural violence, inequality, and power struggles [16,19] (see also [79]). For Cafer et al. [31] and Jennings [32], such stressors are a reflection of the powerful political and economic interests often attached to, and promoted by, calls for a “bounce back” kind of resilience.

Calls for both more and better community engagement are welcome, but we argue that “engagement” is itself a bureaucratic concept and orientation: communities themselves are less interested in “engagement” per se than in addressing community needs. We believe that formal systems and institutions need to support the visions, goals, lived experiences, and on-the-ground expertise of communities, including a willingness to critically interrogate systems of privilege, structural racism, and procedural (in)justice embedded in institutional practices and policies. To be clear, re-centring community does not imply that communities speak with one voice or are inherently wise beyond measure. We acknowledge the concept and operationalization of community has long been contested [80], and can take on communitarian, utilitarian, libertarian, or “geo-anarchist” flavours [81]. Community as an object of interest is often defined by professionals in order to enable “community work” [82], whereas it is arguably the felt sense of community that matters most from the perspective of those implicated [83]. For our purposes, community is spatially anchored in neighbourhoods and also reflective of not only shared values (though we are wary of assumptions that community “speaks with one voice”) but also shared history by virtue of processes of marginalization. We prefer a nuanced understanding of community action to totalizing discourses that proclaim it as a priori virtuous (empowering) or problematic (complicit with neoliberal downloading of responsibility from the state to civil society). Rather, it is about recognizing the wisdom of procedural approaches that enable co-production (of resilience, sustainability, social justice) in ways that respect and build upon the local knowledge and expertise, relationships, needs, and aspirations of communities [67]. This is, fundamentally, a relational view of community resilience building and development that understands that investments in the quality of the social fabric, and linking/bridging social capital, are as essential as investments in physical infrastructure [84,85,86].

## 4. Building Resilience: Re-Centring Community

Recent calls to build community-centred “bounce forward” resilience are rarely reflected in dominant structures, processes, and models of decision-making, in part because calls for improved working relationships between formal institutions and communities have not been accompanied by much in the way of tangible guidance for how to do this. Without clear, actionable guidance from the literature, the top-down, institutionally driven approach to resilience building continues to exclude communities who face the “day to day” impacts of shocks and stressors [38]. To centre the lived realities and expertise of communities, especially Black, Indigenous, and other People of Colour (BIPOC) communities that have been marginalized by current systems of power, new models of decision-making must simultaneously support, resource, and bring together both top-down and bottom-up approaches. In the current resilience literature, such models are limited and fail to address broader questions of equity and procedural justice.

While emerging frameworks for building community-centred resilience call for iterative citizen engagement processes, the stand-alone nature of these processes often fails to integrate existing networks, relationships, and neighbourhood development efforts. Despite formal recognition of the need to connect resilience-building initiatives between civil society actors and formal government agencies and departments, our understanding of how best to operationalize this process is still in its infancy. Citizen involvement in resilience-building activities has also been shown to have the potential to build social capital, enhance capacity for collaboration, increase public safety, reduce property crime and violence, and provide cost-savings to cities and regions [2,6,10,11].

Discussions of top-down and bottom-up approaches to resilience building are intrinsically linked to broader issues of governance. It is often argued that the greater the diversity of actors that participate in and benefit from decision-making processes, the greater the chance that resilience-building activities will be durable, equitable, and, potentially, adaptable to other contexts [2,62]. If community members do not share power and responsibilities with formal institutions throughout the resilience-building process, however, there is a risk that these “community” projects will either exacerbate chronic stressors, inequalities, and political disenfranchisement or be short-lived [87]. As detailed case studies often underscore, the challenges of power sharing in practice are significant [88,89,90,91].

While citizens often want to be deeply involved in resilience-building work [92,93], new, co-created structures, spaces, and processes are desired rather than the “community consultation” spaces typically created by formal institutions [67]. The overarching challenge is not simply short-term mobilization, but the long-term institutionalization of locally driven resilience-building efforts. While emerging frameworks for building community resilience call for iterative citizen engagement, these are often stand-alone processes that are not always well integrated into other community development efforts [94,95,96]. Although communities are central to preparing for, responding to, and recovering from extreme events, insufficient attention has been paid to the power dynamics between community, state, and NGO actors, especially during the immediate response phase of an extreme event [41,97,98,99]. Without considerations of equity, resilience-building efforts may reinforce, rather than reduce, existing vulnerabilities and marginalizations [75]. Fisher and Buckner [98] argue that mainstream models of service delivery in marginalized urban communities focus on achieving pre-defined outcomes rather than on elevating the ideas, plans, and strategies of the community, building local capacity and providing the requisite social infrastructure to promote and support leadership within the community.

To bring about long-term structural change, beyond responding to short-term shocks, long-term social, economic, and political inequality stressors need to be addressed. This point has been the cornerstone of the Toronto Resilience Strategy [67,100]. In this vein, Olsson et al. [101] proposed a framework of adaptive governance built upon shared management and responsibility between residents, community organizations, and government agencies. For Folke [34], social networks are the central “web” that “tie together” adaptive governance systems (p. 262), to be involved at every stage of the resilience-building process. LaLone [19] further highlights the need to involve communities not only in short-term, post-shock responses, but also long-term, pre-shock planning.

However, the challenge of engagement is how to translate local voices into institutional change. This process is largely dependent on whether communities’ lived experience, local expertise, and context are the focal point for inclusive planning, response, and recovery efforts, or whether communities are seen simply as the beneficiary of institutionally led planning and action. At issue are forms of urban governance that emphasize co-production with a wide range of stakeholders (especially affected communities) [102,103,104,105,106] the nature of relational networks that facilitate participatory governance [47,51,107], and the broader local socio-political cultures in which particular arrangements are shaped and embedded [73]. In order for a community to truly be resilient, it is often the formal systems and responses that need to adapt to local contexts.

## 5. Community-Based Organizations and Community-Centred Resilience

As previously noted, recent studies report the need for strong pre-existing institutional relationships, participatory structures, and community and institutions’ knowledge of one another to build strong community–institutional relationships before, during, and following disasters [74,108,109,110,111]. On the institutional side, “deeply ingrained social and political divisions” may drive selective relationships with communities [112] (p. 463). On the community side, Fitzpatrick and Molloy [109] and Graham et al. [74] identified mistrust of institutions and authorities as well as prior government wrongdoing as reasons for why such relationships may fail.

Community-based organizations (CBOs) have been advanced as a way forward in fostering effective community/institutional relationships [113,114,115] but in our view fall short in several key respects. While organizations that are physically located in communities can and do play critical roles in fostering local resilience [19,116], the actual roles they play are many and varied. There is a danger in assuming that just because the organization is located in a community, their mandate and funding includes the kinds of connector roles called for in creating community-centred resilience.

CBOs have been recognized for their potential to act as a “strategic link between community members and government” [114] (p. 329), or a “bridge between universal plans and specific needs” [115] (p. 34), but this analysis is not without its challenges. CBOs are often defined as non-governmental organizations that function to address the needs of the local community [97,114]. CBO is often used as an “umbrella term” to capture the immense diversity of service, relief, and civic organizations [97,99]. The risk is that any “organization”, “group”, “committee”, or “association” is described as a CBO in the literature as long as it is located within the community. Such conceptual ambiguity can prevent the effective identification of characteristics or conditions that contribute to building the right social infrastructure to foster community-centred resilience, including successful, authentic, and intentional relationships between community players and institutions engaged in preparing for, responding in, recovering from, and bouncing forward after major shock events.

In order to make the focus on CBO useful in the discussion of community-centred resilience, distinctions can be made between various types of organizations (Table 1). While in practice, some organizations take on more than one of these identities, exploring their focus, structure, and purpose can go a long way in understanding the ecosystem of players involved in mitigating and addressing ongoing stressors and shocks at the community level.

As can be seen above, community-based organizations are many and varied; they can and do play multiple roles in the event of a shock. It is a very specific type of community-based organization, however, that plays the kind of role that connects civil actors with governments, ensures communication flow across a community, and coordinates the work of various actors for maximum effect. This type of organization, which can be called a community backbone organization or integrator, plays a prominent role in a Connected Community Approach.

## 6. A Connected Community Approach

To address the search for an equitable model for the governance of community-centred resilience, a Connected Communities Approach (CCA) is a novel solution for connecting communities and formal institutions. Unlike many other community interventions, the goal of a CCA is centred around strengthening the social fabric of marginalized communities rather than aiming at a specific predetermined outcome. A CCA is particularly relevant to discussions of community-centred resilience, as it fosters community-led, collaborative responses to systemic stressors, thereby developing the relationships and networks that support a community-centred approach to responding to, recovering from, and bouncing forward after major shock events [117,118].

A CCA is a “complex interconnection of principles and practices that builds from previous community development theories” including asset-based community development, complexity theory, systems theory, and collective impact [85] (p. 4). As a set of principles and practices for community development, a CCA argues that by “intentionally focusing on and strengthening the social connections and networks between and among organizations, these networks can be a catalyst to foment community-based social and economic development” [85] (p. 3). By supporting community building from the bottom up and inside out, a CCA emphasizes the central importance of a community backbone organization as critical social infrastructure that provides an “anchoring point for social network structures across levels and sectors (person, to person, organization to organizations, etc.)” [85] (p. 2).

The CCA emerged over a period of intense on-the-ground community development work in East Scarborough, a marginalized inner suburban community in Toronto, Ontario [85,119]. Although it was not coined a CCA until 2014, the early iterations of CCA resulted in the co-creation of the East Scarborough Storefront [85]. Later referred to as a “community backbone organization”, the East Scarborough Storefront was designed as an innovative “by the community for the community” service hub model in 2000 [119], but it soon became apparent that the implications of this facilitative praxis went beyond improving local access to services. As early as 2011, Cowen and Parlette recognized that:

“*The Storefront is much more than a space for residents to access services, however. It has played a profound role in building community capacity and vision, organizing new initiatives and creating opportunity for connection across the diverse threads of the community*”

[120] (p. 4)

As The Storefront matured, it began forming networks of contributors to the community’s overall wellbeing, including grassroots groups, social service organizations, architects, planners, academics, and municipal actors. Collectively, these players began to recognize the critical gap that The Storefront was filling. The Storefront was iteratively and organically weaving networks to create social infrastructure that both strengthened social fabric at a local scale, and at the same time, intentionally connected the community to public policy actors, capital investment, and social networks that are not necessarily local [120]. This was the genesis of what later became the CCA.

The social infrastructure being created by the local community and supported by a community backbone organization was put to the test in 2012, when a mass shooting (described at the time as the largest in Canadian history) took place in the community [121]. In the wake of this major shock event, dozens of community actors and institutional players participated in a coordinated response which put grassroots leaders at the forefront. Institutional and municipal actors worked with and alongside community efforts, rather than running roughshod over them.

The community was prepared to act in this networked, coordinated way because people and organizations had, over time, invested in creating a connected community including multiple networks and coordinated communication mechanisms. The community was, therefore, able to respond with multiple coordinated actions, facilitating responses among 70 organizations/grassroots groups within days of the incident. This intentional building of networked relationships to address the ongoing stressors associated with poverty, marginalization, and racialization in communities is core to a community’s ability to mobilize effectively in the event of a major shock. Smith [122] suggests that it is working in emergence and making “the locus of work...not in building an institution but in building the community’s ability to voice issues and activate solutions, which places ownership in the hands of the community and creates agency” (p. 2). Thus, by building relationships, the community was able to effectively respond to the major shock event.

Beyond the ability of the community to quickly and effectively respond to the shock event, these coordinated efforts elevated the voices of Black, Indigenous, and newcomer youth to surface ways in which the East Scarborough community could work better together to prevent another tragedy and to prepare for and respond to community “shocks” in the future. In this way, the East Scarborough community bounced forward by raising funds to support youth from Black, Indigenous, and newcomer communities to lead their own initiatives and, alongside organizations, to create the kind of social infrastructure they identified as critical to reducing violence in the community [117,123].

Unlike other community-based organizations, The Storefront’s role in the community is not direct service delivery, but rather to facilitate the creation of a “community social fabric that supports people, organizations, and initiatives to thrive” [124]. In 2012, based on the evidence of The Storefront’s extensive impact on the community it served, staff began the process of articulating what made their approach unique and effective in their community and to explore ways in which their work could be applied to other communities with similar results [125]. From this work, the CCA emerged.

A CCA offers an opportunity to bring together the best of planning, design, academic theory, municipal, provincial and federal strategy, social service interventions, faith community aspirations, and corporate social responsibility and ground them in the authentic goals, aspirations, and realities of grassroots groups and people who have traditionally been at the margins. Unlocking the potential of a connected community requires skill sets not often found in our community-based interventions. These include network weaving, facilitation, knowledge mobilization, and translating across multiple actors both within and outside of the community. Using a CCA to unlock the potential of communities requires an investment of time and resources in local capacity building and social infrastructure, but most of all in the facilitative role required to continually weave together the social fabric that communities need to effectively find local solutions to complex social problems [126].

The role of a community backbone organization in the context of community-centred resilience can not only facilitate local responses to shock events, but at its best can also play the vital role of two-way communication between community and government strategy and action. In their 2015 UK study of connected communities (which aligns with but is distinct from the Connected Community Approach originating in East Scarborough), Parsfield et al. [127] argue that “non-statutory duties of public services must not simply be seen as ‘soft’ extras, but potentially crucial points of collaboration & engagement between state and communities as well as strategic opportunities to prevent greater problems arising from social isolation” (p. 5).

One of the unique features of a CCA is that it does not exclude or seek to replace projects, programs, or other approaches in a community. Rather, it builds on these, using principles and practices that are captured in the CCA’s 10 keys for uncovering the potential of a connected community (Table 2).

## 7. Conclusions

In the face of rising foreseen and unforeseen shocks and stressors in some of our most marginalized and racialized communities, discussions of public health, equity, and sustainability in our cities now include the concept of community resilience. There have been recent calls to go beyond thinking about community resilience from a top-down, “bounce back” perspective, to a community-centred, “bounce forward” approach, which means foregrounding the role of communities in responding to, recovering from, adapting to, and transforming before, during, and after crises, as well as resourcing this work (rather than simply downloading responsibility from the State). Community-centred resilience focuses on community context, local knowledge, networks, and assets to create resilience strategies that work for people often marginalized from formal responses, and especially BIPOC communities. However, despite widespread agreement about the need for such approaches, the community resilience literature provides little guidance (and examples) of how to actually go about this, especially when it comes to weaving stronger relationships between community-focused actors in ways that centre and build on the lived experience and embedded knowledge of residents, and that take into account the ways in which chronic stressors and shock events are interlinked.

In this paper, we introduced a Connected Community Approach (CCA) as a practical framework for centring community in ways that meet the criteria identified as missing in the literature. A CCA offers an opportunity to bring together the best of both community-based planning and action and formal centralized emergency response. The role of the community backbone organization (as distinct from just a CBO, as noted above) is central to a CCA and, in the context of community-centred resilience, can not only facilitate local responses to shock events, but at its best can also play the vital role of two-way communication between community and government strategy and action.

The role of a community backbone organization, also known as a community integrator, is a key area for future study. While the CCA has not yet been formally adopted beyond the East Scarborough neighbourhood where it originated [59], what it lacks in geographic reach it makes up for in experience-informed practice. By codifying the approach, The Storefront has shone a light on a role that groups and organizations in communities take on in variously structured and ad hoc ways in an attempt to foster community-centred resilience. In arguing that the Connected Community Approach responds to a key gap identified in the literature in terms of guidance on how to better connect grassroots community and formal institutions before, during, and after shocks, we are suggesting that the broader applicability of the CCA extends well beyond its origins in the Canadian context. As we hope to have made clear, the burden of investing in, learning from, and fostering resilience at a local level, however, should not be placed solely on the community. In order for a community to truly be resilient, it is often the formal systems and responses that need to adapt to local contexts.

A further dimension requiring urgent attention, one that surfaces regularly in this work, and one that our team is exploring, is the need for a racial justice framework within which to situate community resilience-building work, especially as it concerns work in diverse urban settings involving marginalized and racialized communities, where relations with formal institutions are often even more strained because of institutional racism.

The discourse on community-centred resilience provides an opportunity to strengthen the practical capacity of communities and formal institutions to engage in open-minded, locally driven, connected, and equitable processes. A CCA provides a framework to strengthen the capacity of all players to work together to address entrenched ongoing stressors, and to prepare for, respond to, recover from, and bounce forward after shock events.

## Figures and Tables

**Table 1:** A typology of community organizations by structure and role in community-centred resilience [84,108].

Type of Community Organization	Examples	Governance Structure	Role in Community-Centred Resilience
Community-basedorganizations with governance and decision making that rests outside of the community	Public libraries; public health departments; disaster relief organizations such as the Red Cross	Includes any organization with multiple branches and centralized decision-making	Can act as a conduit between larger systems and communities; often have large community-based facilities that can be leveraged for planning and responding activities; often have reduced autonomy in facilitating community driven decision making, planning, and action
Social service organizations	Foodbanks; employment centres; immigration services; legal aid; counselling centres	Governance can be either local or centralized elsewhere; mandates primarily focus on addressing individual needs	Play critical roles in helping individuals with needs caused by chronic stressors and major shocks are typically focused on the individual/professional relationship rather than on facilitating collective action
Interest focused organizations	Arts organizations; recreational sports leagues; after-school programs	Governance can be either local or centralized elsewhere; mandates primarily focus on convening around shared interests including drama, music, or sports groups	These groups can play specific and even surprising roles in the event of an extreme shock, but are not usually designed to facilitate community-wide processes
Grassroots organizations	Mutual aid networks; peer to peer support groups; residents’ and neighbourhood associations	May or may not have formalized structures; deeply rooted in communities; usually have a purpose/focus on either service delivery, community development, or advocacy	Critical players in community-centred resilience; they often hold knowledge and relationships with community members that formal institutions cannot
Community development organizations	Community Development Corporations	Governance and decision making is firmly in the community with significant grassroots and resident participation. The purpose of these organizations is to foster processes and build local capacity to generate community-led solutions to local issues.	These organizations are critical in ensuring the resilience efforts are truly community centred. Planning and execution of strategies are based on local context, lived experience, and local knowledge. May or may not hold or foster relationships with formalized structures outside of the community.
Community backbone organizations (local integrators or intermediaries)	East Scarborough Storefront (Toronto)	Like community development organizations described above, these organizations have community driven governance and decision making structures. The primary purpose of these organizations is to facilitate connections, strategy and action, between and among the various players engaged in community-building work	These organizations are ideally suited to bridging grassroots, civil society actors and more formalized organizations, institutions, and governments; facilitate processes that allow the various actors to collectively, plan for, respond to, recover from, and bounce forward after major shock events.

**Table 2:** Ten keys for a Connected Communities Approach [84].

Key	Description
Build on everyone’s strengths	CCA is an asset-based approach that emphasizes that local residents are not vulnerable people waiting to be helped, but people with agency to affect the outcomes in their own community. Strengths can be found in local residents, organizations, and physical and natural resources. CCA seeks to nurture local strengths and connect them to the opportunities that emerge both from within and outside the community.
Create connected communities from the inside out	CCA is a practical approach that supports the idea that there needs to be key actors in a community that intentionally focus on strengthening and connecting the actions and initiatives taken on by a diversity of local actors. The entity that plays this role is sometimes called a community backbone organization, an integrator or community facilitator. CCA suggests that whatever the entity is called, it needs to be locally created, governed, and deeply rooted in co-created values and principles and to prioritize a healthy work environment.
Facilitate collaborative processes	CCA is built on relationships. CCA posits that community-centred resilience is about how a multitude of individual actions interact with each other to strengthen the overall social fabric of the community in agile and adaptable ways. Therefore, in a CCA, the community backbone organization focuses on network weaving and strategic facilitation so that community actors and institutions can leverage each other’s strengths and work better together.
Learn together	CCA both embeds learning feedback loops and knowledge mobilization to strengthen a community on an ongoing basis and over time, in the event of a major shock event, accumulated collective experience of intentional and collective learning can help everyone (grassroots groups, institutions, organizations, funders, etc.) generate stories and various cultural ways of knowing to reflect, learn, and adapt together.
Embrace the messiness	CCA likens the community to a natural ecosystem, evolving and adapting over time and changing with the introduction of each new stimulus. CCA emphasizes the capacity for communities to work in emergencies, making their ability to respond and adapt in a crisis much more nimble than a government-led emergency response. CCA focuses on a community’s unique capacity to adapt to ever changing contexts, which is important when addressing ongoing stresses and paramount when responding to, recovering from, and bouncing forward after a shock event.
Prioritize equity and power sharing	Power is a complex dynamic in all communities. Labeling people in marginalized communities as “vulnerable people” maintains the status quo and perpetuates systemic inequities. CCA sees shifting power and locus of knowledge, action, and decision making as fundamental to community-centred resilience.
Let values lead	CCA is a values-driven approach. In using a CCA methodology to bridge differences, success can be measured by the degree to which diverse sets of players can share common purpose and values and draw on their own experiences, passions, and talents to co-design solutions. Designing and implementing purpose- and values-based strategies allows for creativity, innovation, and agility in the face of complex local challenges (both shocks and stressors).
Work at multiple scales	Community-centred resilience does not mean downloading responsibility for emergency response to communities. Instead, it means strengthening both community-led response and government actions by intentionally investing in the connections between the two. Unlike other CBOs and community development strategies (see Table 1), CCA focuses on both building strong local social fabric, and on connecting community-led initiatives to larger systems, thereby simultaneously centring community and taking full advantage of the knowledge, resources, and opportunities afforded by the scale of larger systems.
Make community building visual	Communication is foundational to community-centred resilience. How communication flows within a community and between community and governments/institutions can make or break local response or recovery efforts. CCA focuses on using creative and visual ways to mobilize knowledge and facilitate effective local communication channels. This means that, in a shock event, local people know where to go to receive trusted information, how to shape the response undertaken, and share knowledge effectively with their networks.
Build creative infrastructure	CCA is predicated on the imperative to invest in the kinds of social infrastructure that strengthens local decision making, agency, and influence on broader systems. A connected community requires intentional structures to ensure that the community really does strengthen over time and can effectively respond to, recover from, and bounce forward after major shock events. In CCA, creative infrastructure means putting as much emphasis on investing in the supports, facilitative roles, and connective tissue that centre community priorities and actions as on the buildings and structures in which those activities take place.
